# A Rolling Bearing Fault Classification Scheme Based on k-Optimized Adaptive Local Iterative Filtering and Improved Multiscale Permutation Entropy

**DOI:** 10.3390/e23020191

**Published:** 2021-02-05

**Authors:** Yi Zhang, Yong Lv, Mao Ge

**Affiliations:** 1 Key Laboratory of Metallurgical Equipment and Control Technology, Wuhan University of Science and Technology, Ministry of Education, Wuhan 430081, China; yizhang_de@163.com (Y.Z.); ge1656372625@gmail.com (M.G.); 2 Hubei Key Laboratory of Mechanical Transmission and Manufacturing Engineering, Wuhan University of Science and Technology, Wuhan 430081, China

**Keywords:** permutation entropy (PE), k-optimized adaptive local iterative filtering (ALIF), improved multiscale permutation entropy (improved MPE), BP neural network, fault classification

## Abstract

The health condition of the rolling bearing seriously affects the operation of the whole mechanical system. When the rolling bearing parts fail, the time series collected in the field generally shows strong nonlinearity and non-stationarity. To obtain the faulty characteristics of mechanical equipment accurately, a rolling bearing fault detection technique based on k-optimized adaptive local iterative filtering (ALIF), improved multiscale permutation entropy (improved MPE), and BP neural network was proposed. In the ALIF algorithm, a k-optimized ALIF method based on permutation entropy (PE) is presented to select the number of ALIF decomposition layers adaptively. The completely average coarse-graining method was proposed to excavate more hidden information. The performance analysis of the simulation signal shows that the improved MPE can more accurately dig out the depth information of the time series, and the entropy value obtained is more consistent and stable. In the research application, rolling bearing time series are decomposed by k-optimized ALIF to obtain a certain number of intrinsic mode functions (IMFs). Then the improved MPE value of effective IMF is calculated and input into backpropagation (BP) neural network as the feature vector for automatic fault identification. The comparative analysis of simulation signals shows that this method can extract fault information effectively. At the same time, the experimental part shows that this scheme not only effectively extracts the fault features, but also realizes the classification and identification of different fault modes and faults of different degrees, which has a certain application prospect in the research and application direction of rolling bearing fault identification.

## 1. Introduction

The normal operation of key mechanical equipment is an important guarantee for industrial production, especially the running state of the rolling bearing in mechanical equipment [1,2]. Its operating state is closely related to the stable operation of the equipment, so its fault diagnosis technology is particularly important. In the fault diagnosis of mechanical equipment, fault feature extraction and pattern recognition are the key steps [3,4,5]. The results of feature extraction usually represent the extracted state feature parameters. Pattern recognition is essentially a process of comparison and classification [6,7]. It judges fault types by comparing current fault features with standard or existing fault features [8,9].

Vibration signal analysis is a common means of fault diagnosis [10]. When the fault diagnosis of mechanical equipment is carried out, the vibration signal can be extracted and the effective information reflecting the fault characteristics can be separated [11]. However, in practical applications, the vibration signals of rolling bearings collected often have strong nonlinear characteristics, while the traditional analysis methods are usually based on the assumption of signal stability, and non-stationary signals in the time and frequency domains cannot be considered at the same time [12,13]. Therefore, they have certain limitations. In recent years, the commonly used nonlinear analysis methods mainly include short-time Fourier transform (STFT), wavelet transform (WT), empirical mode decomposition (EMD), ensemble empirical mode decomposition (EEMD), local mean decomposition (LMD), among others. However, STFT [14,15] is difficult to find a suitable short-time window function, so that the signal satisfies the stationarity assumption at a given time width, and does not make the window function too narrow. Especially when the frequency of the signal changes rapidly, its analysis effect is not ideal. WT [16,17] is theoretically more complete than STFT, but the result of wavelet analysis depends greatly on the choice of wavelet basis function, so the difficulty lies in choosing the appropriate wavelet basis function. EMD [18,19] can decompose the time series into several single-frequency modes according to the signal itself. However, EMD is highly dependent on extremum searching method, carrier-envelope interpolation, and termination conditions, which makes EMD have endpoint effect, mode aliasing, decomposition stop criteria, and other problems. In addition, EMD lacks a strict mathematical theory foundation and has poor noise resistance. Wu et al. proposed EEMD [20,21] based on a noise-assisted approach. The principle is to add appropriate white noise to make it continuous on the time scale, which can better separate the inherent scale of the signal. But it still has the phenomenon of mode overlapping and large decomposition error. LMD [22,23] is an improved method in EMD. Its main purpose is to decompose the time series into a finite number of product functions (PFs), and the instantaneous frequency of each PF represents a certain characteristic of the original signal. By combining the instantaneous frequency and instantaneous amplitude of each PF component, the time-frequency distribution of the original signal can be completely expressed. However, the moving average algorithm used in the LMD method for calculating the local mean curve and the envelope curve needs many iterations, so it has a large amount of computation and low computational efficiency.

Recently, Cicone et al. [24,25,26,27] proposed adaptive local iterative filtering (ALIF) method based on the Fokker–Plank equation, which can also filter the signal into several sums of IMFs. To realize the adaptive selection of filter function, the filter function with adaptive characteristics is constructed according to the basic solution system of the Fokker–Plank equation in different filter spaces, which makes the algorithm have a solid mathematical foundation so that the filter interval can change according to the change of filter function to achieve the purpose of adaptive decomposition. In addition, this method can effectively avoid modal aliasing, and significantly overcome the lack of theoretical basis in EMD [28]. According to its theoretical research, the ALIF method needs to preset the number of decomposition layers [29]. The value of the decomposition layer affects the pros and cons of the final decomposition result. If it is too small, the information of the decomposed modal component will be lost. If the value is too large, the decomposition and operation amount will be increased, and the decomposition effect of the original signal is not good. Therefore, how to choose the appropriate k value before decomposition is the key to the wide application of the ALIF algorithm. Permutation entropy (PE) is presented by Bandt [30], which is used to calculate the irregularity and complexity of nonlinear signals. The method is fast and convenient to calculate, has good anti-noise performance, strong robustness, and can detect sudden changes of signals [31,32,33]. When the signal is decomposed by ALIF, the nonlinear factors and complexity of each mode are different, so the PE value is different. By setting the threshold of permutation entropy, we can judge whether there is an abnormal component in the signal to achieve k-value optimization.

Complexity is a non-linear characteristic reflecting the essence of a complex system, and the complexity of signal is different in different states [34,35,36]. The IMFs generated by the ALIF method contains the fault feature information of the original signal. To quantify these fault features, the entropy theory is introduced. The PE mentioned above can be used for calculating the uncertainty of the time series. However, the output time series of the complex system contains characteristic information on multiple scales [37,38]. Multiscale permutation entropy (MPE) is defined as the entropy of permutation at multiple scale factors, which can effectively obtain the vibration information of vibration signals at multiple scale factors and effectively characterize the random mutational behavior of time series compared to single scale permutation entropy. However, the time series coarsening process leads to shorter time series as the scale factor increases in the MPE algorithm, which inevitably causes the lack of feature information of the vibration signal at larger scales. For this reason, this paper improves the multiscale coarse-grained time series and proposes an improved multiscale permutation entropy (improved MPE). The entropy value is calculated for each coarse-grained sequence, and then the entropy value of the obtained coarse-grained time series is averaged as the final eigenvalue. This process greatly optimizes the inadequate coarse-grained process in the MPE algorithm and makes the average improved MPE value obtained from multiple coarse-grained sequences less dependent on the length of the time series, and better preserves the rich feature information contained in the vibration signal at multiple scales. For the rolling bearing signals collected by the actual sensor, the dynamic characteristics of different fault types and different fault degrees are different, resulting in different signal complexity [39]. The improved MPE analysis of the modal components with rich information can better reveal the fault complexity of the rolling bearing. In addition, the performance of MPE and improved MPE are compared. As can be seen from the results, the improved MPE is smoother in feature extraction and less error in extracting multiple samples.

After fault extraction using improved MPE, the characteristics of multiscale entropy are employed as a feature vector. Then, the obtained feature vectors are imported into the backpropagation (BP) neural network to distinguish rolling bearings with different fault types and different fault levels, which can identify the BP neural network has good generalization ability and strong self-adaptation and self-learning ability [40,41,42,43]. The feature extraction performance of k-optimized ALIF is verified by simulation signals. The subsequent experiments illustrate the feasibility of fault identification of the proposed method.

The following gives the remaining organizational structure: Section 2 introduces the model decomposition process, the basic principles of ALIF, and its optimization process. In Section 3, MPE and improved MPE are introduced respectively, and their performances are compared and analyzed. Section 4 uses the k-optimized ALIF method to process the simulated signals and compare them with EMD and EEMD respectively. Section 5 validates the proposed method and compares it with several similar methods. The conclusion and research direction are given in Section 6.

## 2. Theoretical Description

### 2.1. Adaptive Local Iterative Filtering

EMD can adaptively decompose the fluctuation and trend of different scales in the signal s(t), and can obtain several intrinsic mode functions (IMFs) $u_{i}(i=1,2,\ldots ,Q)$ and a residuals z(t):

(1) $$s(t)=\sum_{i=1}^{Q}u_{i}(t)+z(t)$$

The IMF obtained by decomposition must meet the following conditions:

(1) Over the entire signal length, the number of extreme points and the number of zero crossings must differ by one or the same.
(2) The average value of the obtained upper envelope and lower envelope is zero.

In the process of EMD, performing cubic spline interpolation on the obtained envelope is easily affected by high-frequency noise and causes mode aliasing. Based on empirical mode decomposition, iterative filtering uses convolution instead of an envelope, and its moving average operator is:

(2) $$\xi (s(t))=s(t)*p(t)=\int_{-l}^{l}s(t+\tau )p(\tau )d\tau$$

where p(t) is a low-pass filter and satisfies $\int_{-l}^{l}p(\tau )d\tau =1$, and l is mask length.

ALIF has made two improvements based on iterative filtering (IF). First, it can adaptively calculate the filter length, then Equation (2) can be written as:

(3) $$\xi (s(t))=s(t)*p(t)=\int_{-l(t)}^{l(t)}s(t+\tau )p(t,\tau )d\tau$$

(4) $$\int_{-l(t)}^{l(t)}p(t,\tau )d\tau =1$$

where p(t,τ),τ∈[−l(t),l(t)] is the filter at time t, and l(t) is the variable mask length. The first intrinsic mode function is obtained through the screening process:

(5) $$u_{1}(t)=\underset{n\to \infty }{\lim}\xi_{1,n}(s_{n}(t))$$

where $s_{n}(t)=\xi_{1,n-1}(s_{n-1}(t))$,$s_{1}(t)=s(t)$, and the remaining signal is $z(t)=s(t)-u_{1}(t)$. Repeat the above steps to obtain the remaining intrinsic mode functions:

(6) $$u_{q}(t)=\underset{n\to \infty }{\lim}\xi_{q,n}(s_{n}(t))$$

Too much repeated screening is easy to make IMF become a constant amplitude FM signal, thus losing its physical significance. Therefore, the following termination criteria are adopted:

(7) $$\frac{{\left\|\xi_{i,n}-\xi_{i,n-1}\right\|}_{2}}{{\left\|\xi_{i,n-1}\right\|}_{2}}\leq \varepsilon$$

When the convergence criterion is satisfied, it is set as the intrinsic mode function, in which ε is the preset parameter.

Another improvement is the adaptive calculation of the filter function. Based on the basic solution system of FP differential equations in different filtering intervals, the adaptive FP filtering function is constructed to prevent the local distortion of iterative filtering when processing nonlinear and non-stationary signals.

### 2.2. K-Optimized ALIF Based on PE

In the traditional ALIF algorithm, due to the theoretical limitations of the algorithm, the user needs to set threshold parameters and decomposition levels before signal processing. It is found that the number of decomposition layers will affect the decomposition results, and the excessive decomposition scale may make it difficult for components to express the local features of the signal. To adaptively select the number of decomposition layers, this paper proposes an adaptive selection algorithm based on PE. The purpose of this algorithm is to calculate the PE value of each layer IMF obtained from the decomposition of the original signal, and judge whether the signal has been over decomposed according to the threshold value of PE value. 

Therefore, after setting the threshold value $H_{p}$ of PE, determine the value of IMF in each layer of decomposition result. Whether the permutation entropy is less than the threshold $H_{p}$ can determine whether the signal has over decomposition [44]. The algorithm flow of k-optimized ALIF is as follows:

Step 1. Set the initial value of the decomposition layer k and $H_{p}$ the threshold of the PE to 2 and 0.2 respectively.

Step 2. The measured signal is decomposed by the ALIF algorithm to obtain k intrinsic mode functions $imf_{i}(t)\;(i=1,\cdots ,k)$.

Step 3. Calculate the permutation entropy $pe_{i}(i=1,\cdots ,k)$ of each IMF after decomposition.

**Figure 1:**
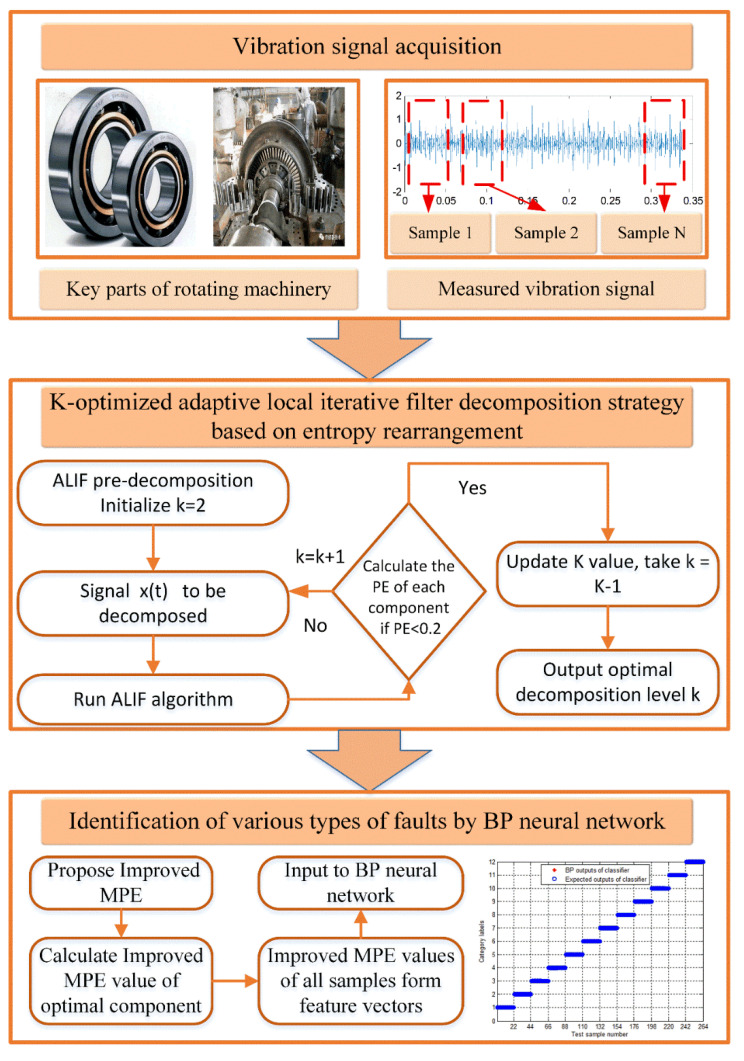
The flow chart of the scheme proposed in this paper.

Step 4. Determine whether $pe_{i}$ is less than $H_{p}$. If it is satisfied, it indicates that excessive decomposition occurs, stop the loop, take k=k−1, and proceed to step 2. If it is not satisfied, the number of decomposition layers needs to continue to increase, let k=k+1, from step 2, continue to perform ALIF decomposition of the original signal according to the updated k value. The algorithm flow is shown in Figure 1.

## 3. Improved Multiscale Permutation Entropy

### 3.1. Multiscale Permutation Entropy

PE can be served to describe the complexity of the system. Compared with the similar complexity parameters such as the Lyapunov exponent and fractal dimension, it is simple in the calculation and good in anti-interference effect and can capture small changes in the system. For complex systems, PE may miss some useful information. To better represent more information of time series, it is necessary to perform multiscale permutation entropy (MPE) analysis on time series. The calculation steps are as follows:

Step 1. The time series $s_{i},\;i=1,2,\ldots \ldots ,L$ with L length is coarsened to get the coarsening sequence $y_{n}^{\left(\tau \right)}=\frac{1}{\tau }\sum_{i=(n-1)\tau +1}^{n\tau }s_{i}\;\;\;n=1,2,\ldots \ldots ,[L/\tau ]$, where τ is the scale factor and [L/τ] is the integer of L/τ.

Step 2. Time reconstruction of $y_{n}^{\left(\tau \right)}$ is performed to obtain $Y_{l}^{\left(\tau \right)}=\{y_{l}^{\left(\tau \right)},y_{l+\tau }^{\left(\tau \right)},\ldots ,y_{l+(m-1)\tau }^{\left(\tau \right)}\}$, where m represents the embedding dimension, τ represents the delay time, and l is the l-th reconstruction component l=1,2,…,L−(m−1)τ.

Step 3. By arranging the time reconstruction sequence in ascending order, the symbol sequence $S(g)=\{l_{1},l_{2}\ldots \ldots ,l_{m}\}$ can be obtained. where g=1,2,…,R and R≤m!. Calculate the probability $p_{g}$ of the occurrence of each symbol sequence.

Step 4. The PE of the coarse-grained sequence is obtained by the following equation, and thus the PE of the time series at multiple scales is obtained.

(8) $$H_{p}(m)=-\sum_{g=1}^{R}P_{g}\ln P_{g}$$

When $P_{g}=1/m!$, the maximum value of $H_{p}(m)$ is ln(m!); and it is normalized with $H_{p}=H_{p}(m,\tau )/\ln(m!)$.

**Figure 2:**
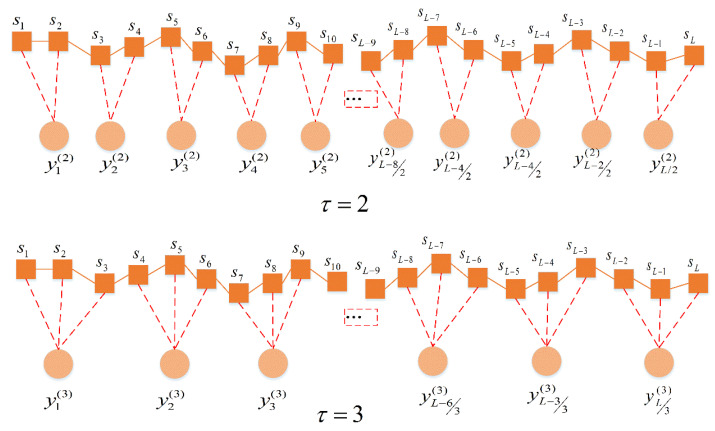
The multi-scale way with scale factors of 2 and 3.

The scale factor is τ and the degree of coarsening is determined by the scale factor. When τ=1, no coarse granulation is performed. Therefore, the multiscale permutation entropy also degenerates into the sequence of permutation entropy. Figure 2 illustrates the algorithm of coarsening process with the scale factor τ=2 and τ=3 as examples.

### 3.2. Improved Multiscale Permutation Entropy (Improved MPE)

The coarse-graining method in MPE may miss some scale information. To overcome the shortage of coarse-graining in MPE, this paper proposes an improved MPE. Its calculation steps are as follows:

Step 1. The coarse-grained sequence is obtained for the time series s(t)  (t=1,2,…,L) of length L to obtain the coarse-graining sequence $y_{k}^{\left(\tau \right)}=\{y_{k,1}^{\left(\tau \right)},y_{k,2}^{\left(\tau \right)},\ldots ,y_{k,\tau }^{\left(\tau \right)},\}$.

Where $y_{k,j}^{\left(\tau \right)}=\frac{1}{\tau }\sum_{i=(j-1)\tau +k}^{j\tau +k-1}s_{i}\;\;\;$$\begin{array}{l} j=1,2,\ldots ,[L-\tau /\tau ]\\ k=1,2,\ldots ,\tau \end{array}$.

Step 2. For each scale factor τ, the PE of each coarse-graining sequence $y_{k}^{\left(\tau \right)}$ is calculated, and then the improved MPE $P_{improved\;MPE}$ is obtained by averaging τ entropy values.

(9) $$P_{improved\;MPE}(s,\tau ,m,t)=\frac{1}{\tau }\sum_{k=1}^{\tau }PE(y_{k}^{\left(\tau \right)},m,t)$$

**Figure 3:**
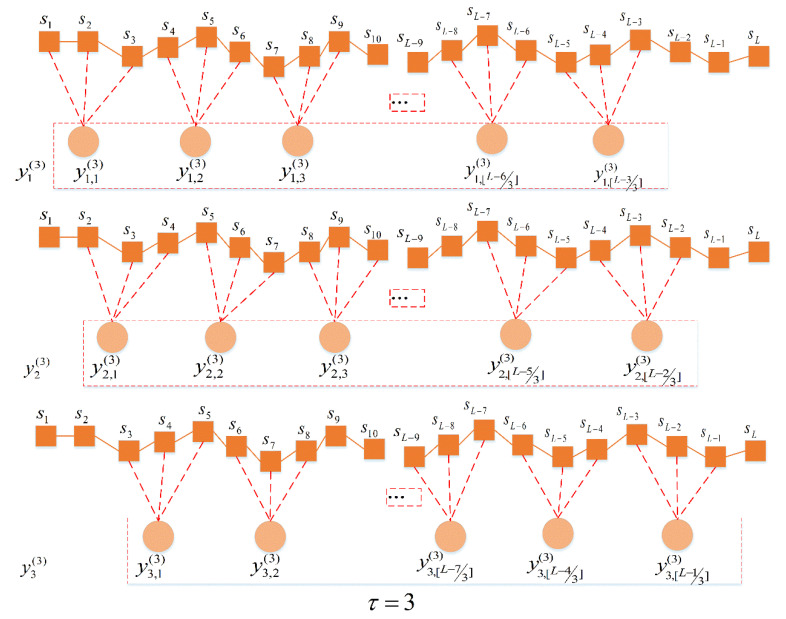
The multi-scale way with scale factor equaling to 3.

Theoretically, improved MPE takes into account information on all τ coarse-grained sequences with a scale factor of τ, and can extract more information than MPE’s single coarse-grained sequence, thus avoiding the entropy fluctuation caused by a single coarse-grained sequence. Therefore, compared with the MPE curve, the improved MPE curve changes more smoothly with the increase of scale factor. When the scale factor is 3, the coarsening process is shown in Figure 3. 

### 3.3. Performance Comparison between MPE and Improved MPE

**Figure 4:**
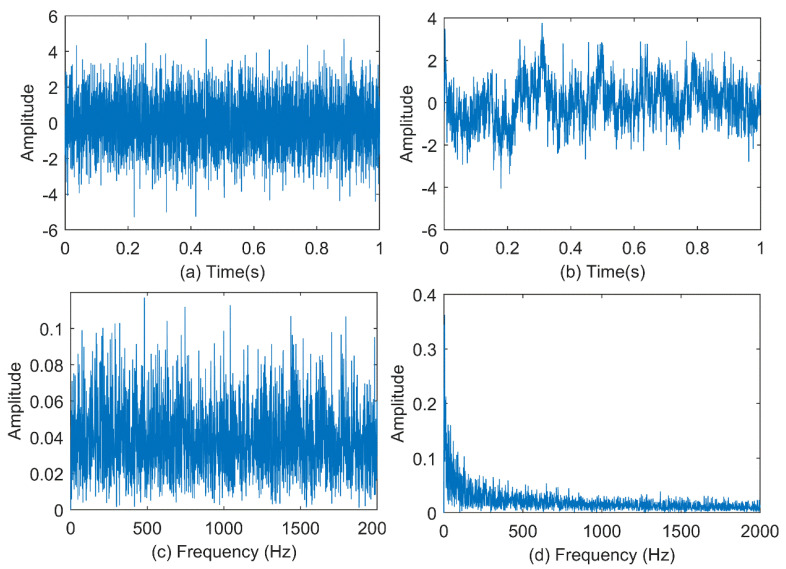
Gaussian white noise and 1/f noise waveform and its spectrum: (**a**) Sequence of gauss white noise; (**b**) Sequence of 1/f noise; (**c**) Frequency domain of white noise; (**d**) Frequency domain of 1/f noise.

**Figure 5:**
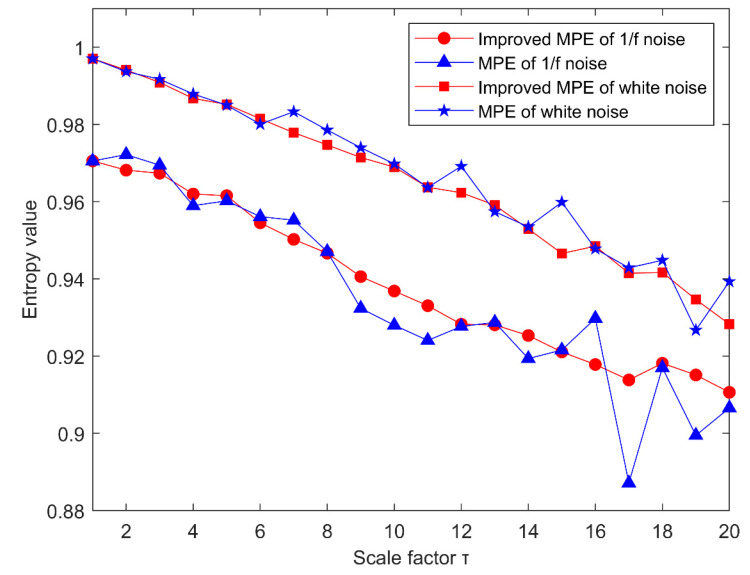
MPE and Improved MPE analysis of Gauss white noise and 1/f noise.

To illustrate the effectiveness of the improved MPE method, the following two signals are used to compare the improved MPE method and the MPE method: white noise and 1/f noise. The time series of 4096 points are drawn in Figure 4a,b. At the same time, the corresponding spectrum is shown in Figure 4c,d. From their spectrum, we can conclude that the white noise spectrum is very uniform and the amount of information contained will be very small, while the frequency amplitude of the 1/f noise spectrum decreases from low frequency to high frequency in turn, so it contained more information than white noise. Firstly, improved MPE and MPE are used to analyze the above signal with a scale factor of 20. The results are shown in Figure 5. It can be concluded that the permutation entropy curve obtained by the improved MPE method is smoother and more stable. Then we can get the following conclusions. The improved MPE method is more stable than the traditional MPE method in analyzing the complexity of signals.

**Figure 6:**
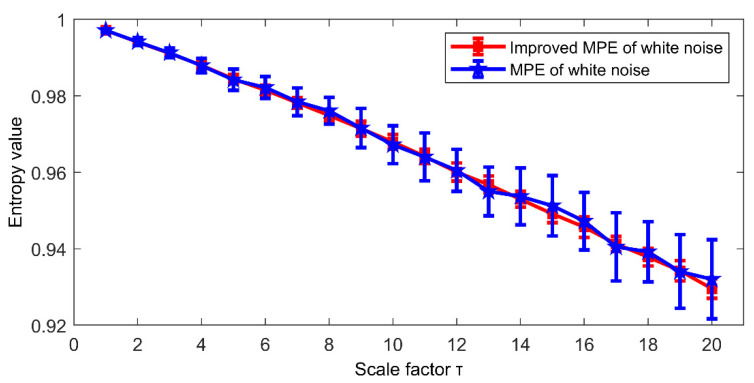
The mean curve and error bar of white noise calculated by MPE and improved MPE.

**Figure 7:**
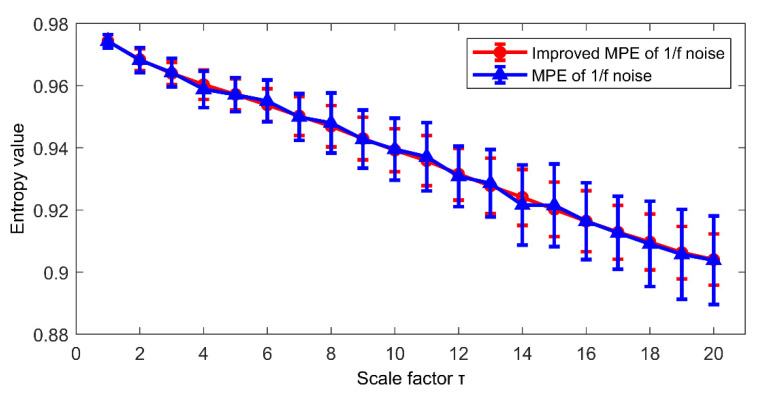
The mean curve and error bar of 1/f noise calculated by MPE and improved MPE.

Then, to further study the estimation performance of improved MPE and MPE, 50 groups of the above two noises were taken for analysis. The mean value curves and error bars of 50 sets of arrangement entropy for each scale factor of 2 kinds of noises are shown in Figure 6 and Figure 7, respectively. It should be noted that the error bar is derived from the standard deviation of the entropy values. It can be obtained from Figure 6 that the mean curve fluctuation calculated by MPE is greater than the trend of improved MPE, and the error bar calculated by improved MPE is much smaller than that obtained by MPE. For the 1/f noise in Figure 7, the error bar of improved MPE is smaller than that of MPE, although the trend of the mean curve obtained by using MPE and improved MPE is basically the same. The above results show that improved MPE has a better application in computing the complexity of time series, especially in computing the results of multiple data sets. 

## 4. Numerical Simulation Analysis

When the bearing fails, the measured signal always consists of a modulation signal, harmonic signal, and noise. To research the feasibility and validity of this method, the following fault signal model is constructed to simulate the running condition of the rolling bearing:

(10) $$\begin{array}{l} x1=\sin(2\pi f_{1}t)(1+\cos(2\pi f_{2}t))\\ x2=\sin(2\pi f_{3}t)\\ f=x1+x2+n\left(t\right) \end{array}$$

**Figure 8:**
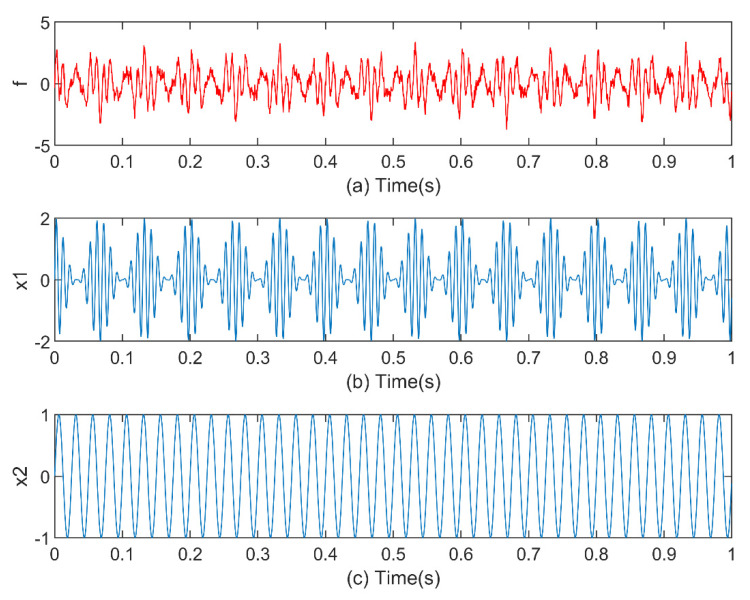
Time-domain waveforms of simulation signal f and its components.

In the formula, the frequencies $f_{1},f_{2},f_{3}$ are sequentially 100 Hz, 15 Hz, and 40 Hz. The sampling frequency is 2048 Hz and the sampling time is 1 s, and n(t) is Gaussian white noise with a standard deviation of zero, as shown in Figure 8.

**Figure 9:**
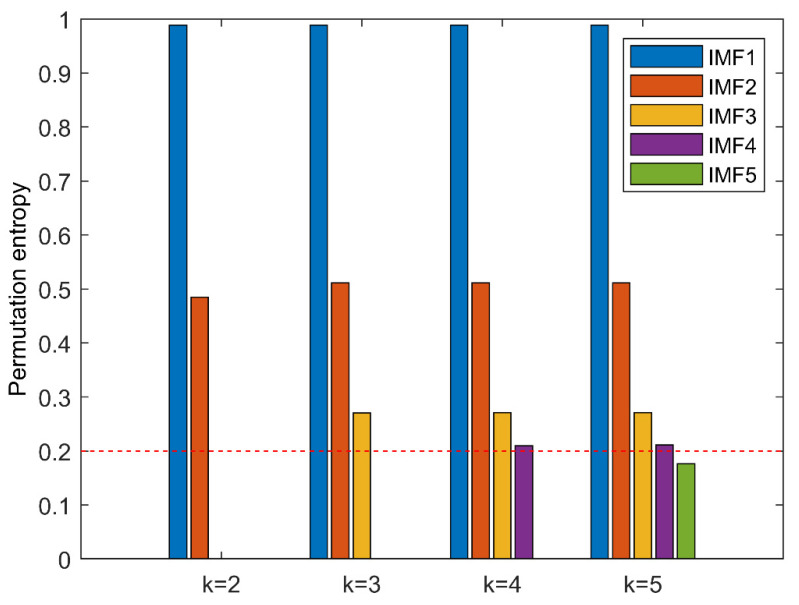
Permutation entropy of each intrinsic mode function.

Firstly, the PE algorithm is used to obtain the decomposition layers, and the initial decomposition layers are set to 2. Cycle iteration is carried out according to whether decomposition has occurred, and the optimal value of k is found. In each iteration, the original signal is decomposed to obtain the PE value of each layer component, as shown in Figure 9. When k = 5, there is a component whose entropy value is less than the threshold value $H_{p}$, but when k = 4, there is no abnormal component. It means that when k = 5, over decomposition happens at this time, therefore the decomposition level of ALIF is selected as k = 4.

**Figure 10:**
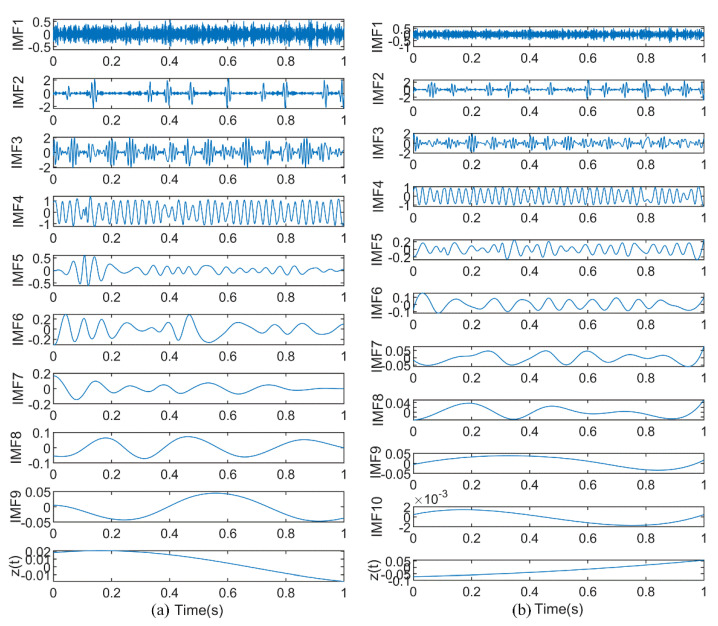
The results of simulation signal f decomposed by (**a**) EMD and (**b**) EEMD.

**Figure 11:**
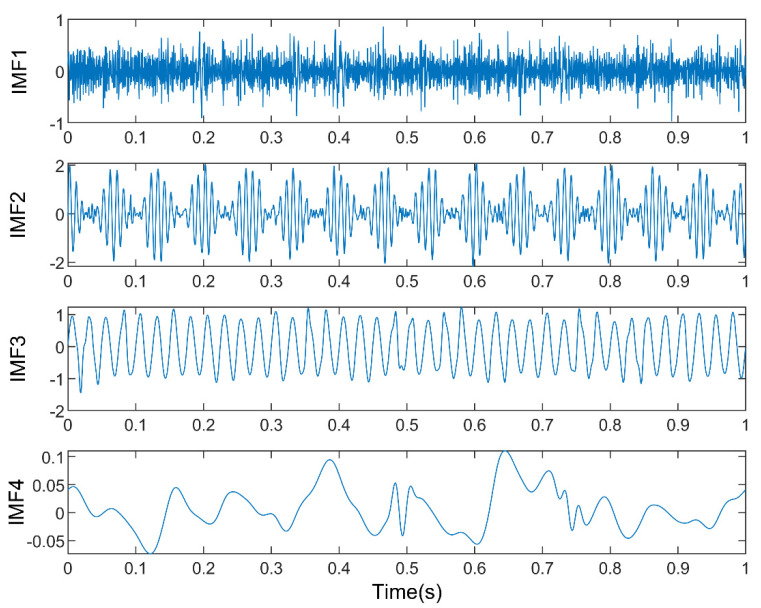
The results of simulation signal f decomposed by k-Optimized ALIF.

To illustrate the decomposition effect of this method, EMD and EEMD are also used to process the time series when the method in this paper is used for analysis. The decomposition results are shown in Figure 10. Figure 10a shows that EMD breaks down the time series into 9 IMFs and 1 residual. Compared with the original signal components, EMD results contain more useless components and the decomposition results are not ideal. Similarly, the processing result of EEMD includes 11 IMFs, as shown in Figure 10b. Compared with EMD, the component obtained by the EEMD method is better than EMD, but it produces more false components. The result of the k-optimized ALIF analysis are shown in Figure 11. The results show that this method has good extraction and decomposition effects on the original components. According to the above comparative analysis, k-optimized ALIF has better stability in modulating signals. 

**Figure 12:**
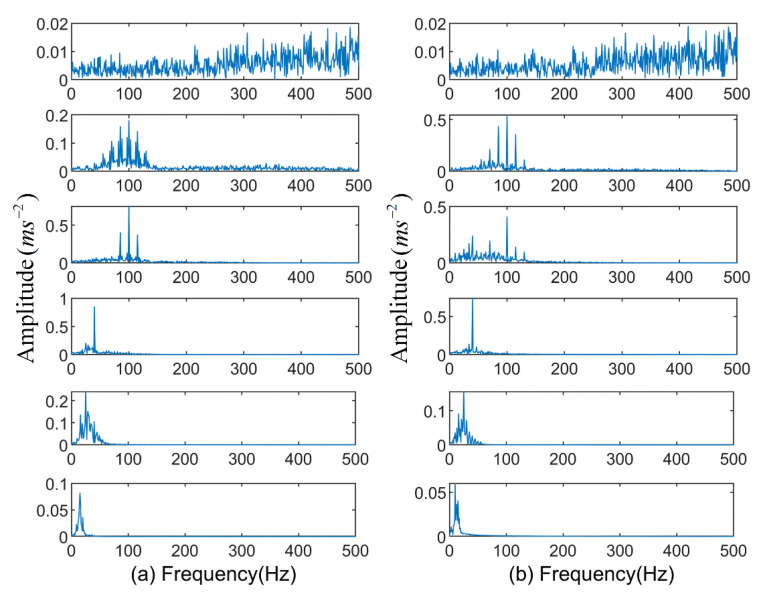
Spectrograms of the first 6 IMFs of the two methods: (**a**) EMD; (**b**) EEMD.

**Figure 13:**
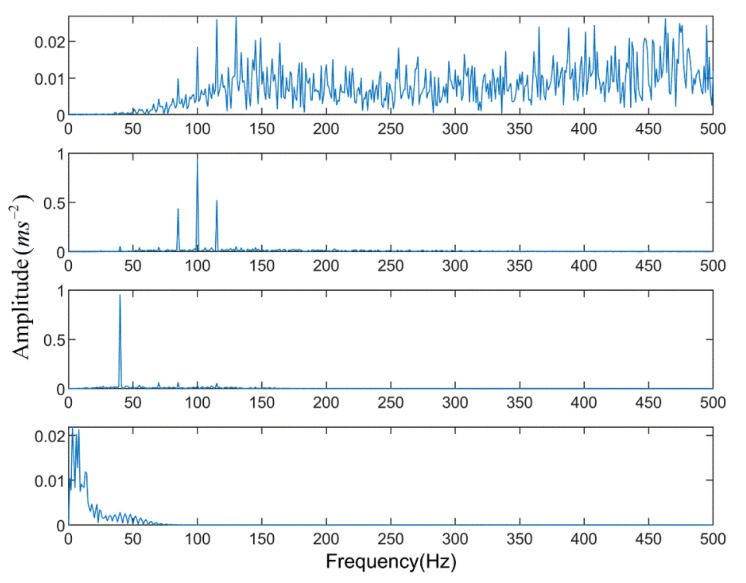
The spectrum was obtained by k-Optimized ALIF.

For further analysis, spectrum analysis is performed on the above components. Figure 12 shows the analysis results of the first 6 IMF components of EMD and EEMD. In Figure 12a, the second and third components contain similar frequency components and their frequency components are similar to the modulated signal, indicating that modal mixing occurs when the EMD processes signals containing modulated components. Similarly, the second and third components in Figure 12b contain similar frequency components, which indicates that the mode aliasing is also generated by the EEMD decomposition method. The spectrum of k-Optimized ALIF is shown in Figure 13. Among them, the frequency of the second and third components are clearly visible and consistent with the frequency of the original signal. It shows that the performance of this method in fault feature extraction is better than that of EMD and EEMD.

## 5. Experimental Study

**Figure 14:**
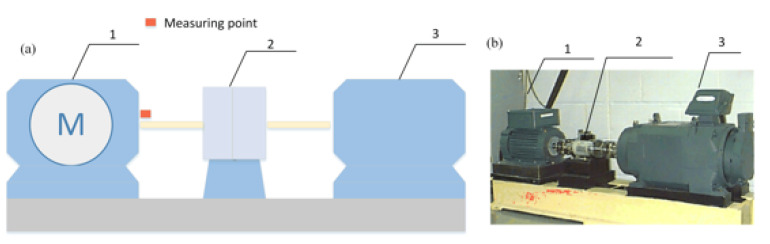
(**a**) Transmission diagram and (**b**) its experimental equipment. 1, 3-Hp motor, 2-Torque transducer, and encoder.

This paper selects the bearing data collected by the website of Case Western Reserve University Bearing Data Center for analysis. The test platform is shown in Figure 14. The acceleration sensors are respectively arranged on the fan end (FE), drive end (DE), and base (BA) of the motor, and the signal acquisition device is a 16-channel DAT recorder. The sampling frequency is set to 12 kHz and the rotation speed is 1797 rpm. The experiment simulated the inner ring fault, outer ring fault, and rolling element fault respectively, and the fault diameter of each fault type includes 0.007 inches, 0.014 inches, 0.021 inches, and 0.028 inches. The experimental failure bearing model is 6205-2RS JEM SKF, each of which, the item parameters, are given in Table 1.

The collected data are truncated by 4096 points for further processing. This paper uses the data when the load is 0 HP for the next analysis. All data types are given in Table 2.

The above vibration signal contains three types of faults and different severity, so this fault classification is actually a twelve-level identification problem. Each category is cut off with 4096 data points, and 29 sets of samples can be obtained, so there are 12 × 29 samples in total. Among them, 7 samples are randomly selected from each category, a total of 12 × 7 samples are used as the training set, and the test set is the remaining 12 × 22 samples.

**Figure 15:**
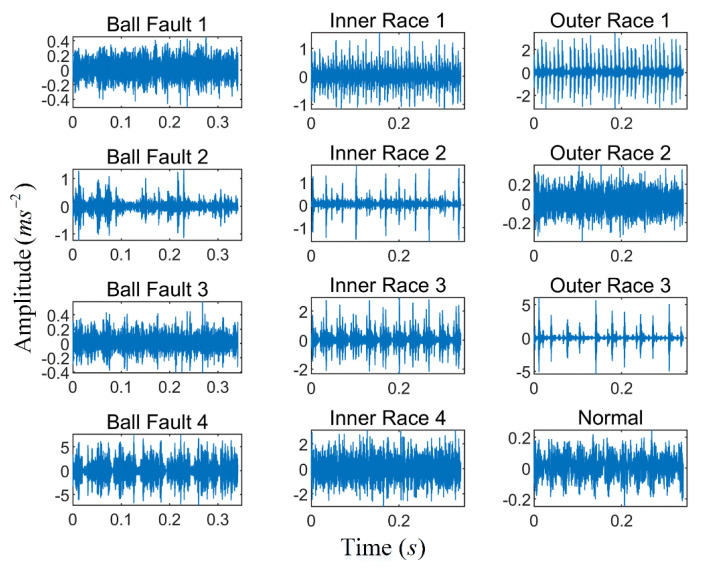
Vibration signals of twelve bearing conditions.

**Figure 16:**
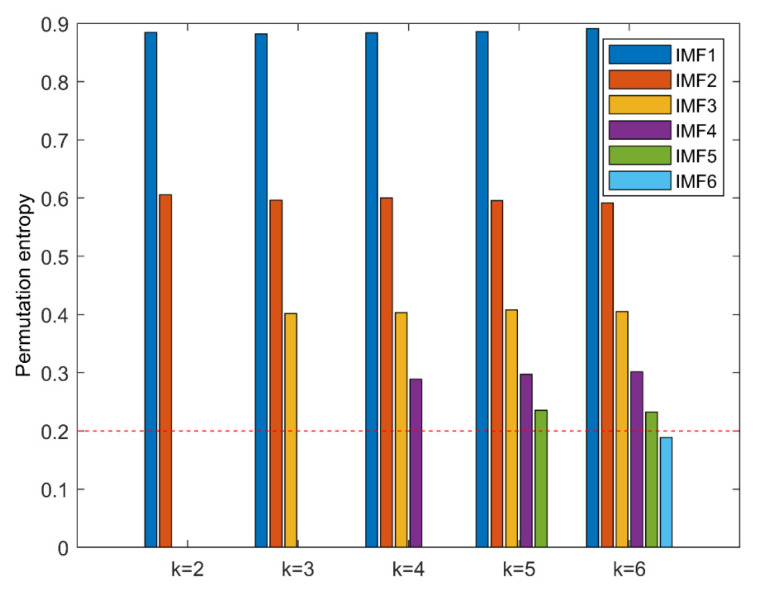
The permutation entropy of IMF of each order after ALIF decomposition.

**Figure 17:**
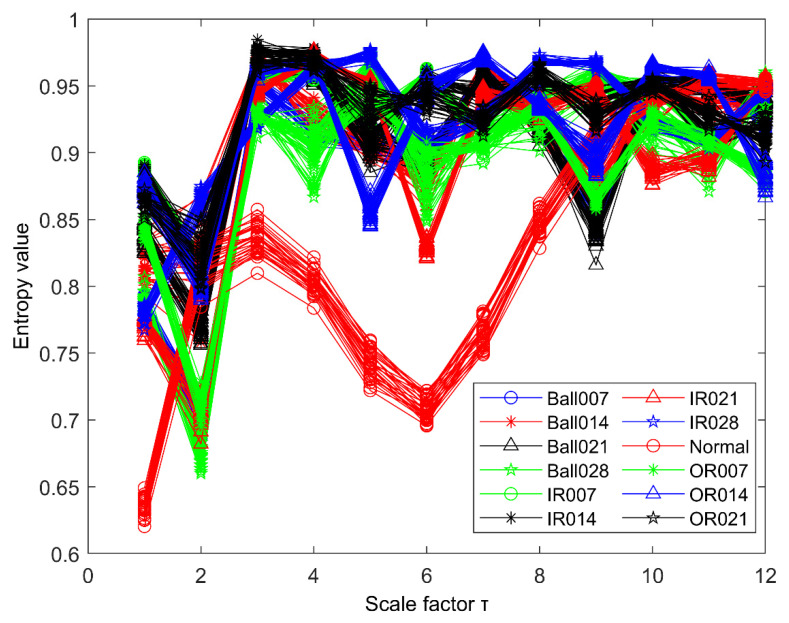
Vibration signals of twelve bearing conditions.

The time series of taking one sample for each group category is shown in Figure 15. It is almost impossible to judge the running state of the bearing only from the time series. Therefore, the original vibration signal must be further processed. Then using the k-optimized ALIF method to deal with it. Similarly, the number of decomposition layers is obtained by PE. As shown in Figure 16, when k = 6, a PE value lower than the selected threshold appears, and when k = 5, there is no abnormal component, so the number of decomposition layers of ALIF is selected as k = 5. According to the k-value optimization process, the component with the most fault features is selected to calculate the improved MPE. Therefore, 12 × 29 sets of improved MPE can be obtained from the decomposition results. Figure 17 shows the improved MPE for all samples under 12 typical operating conditions. As can be seen from Figure 17, the time series of 12 categories have different entropy values at different scales.

**Figure 18:**
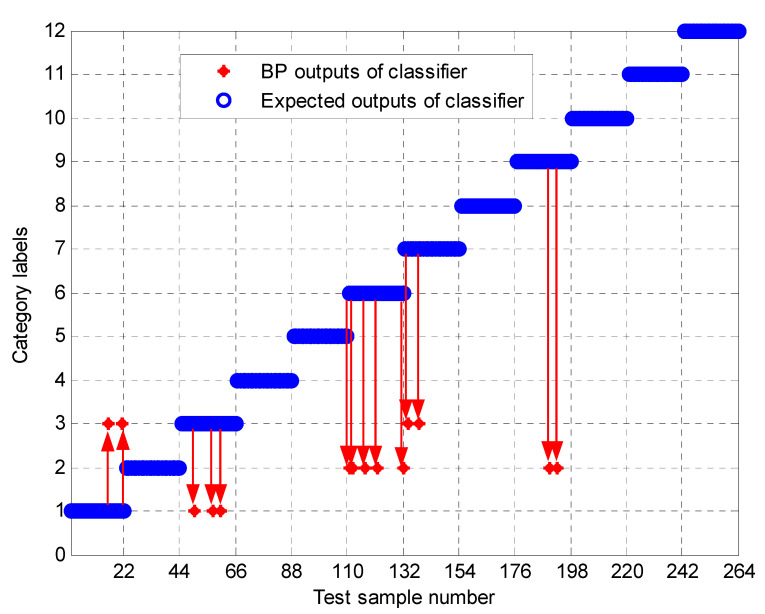
Output of the MPE-based methods.

**Figure 19:**
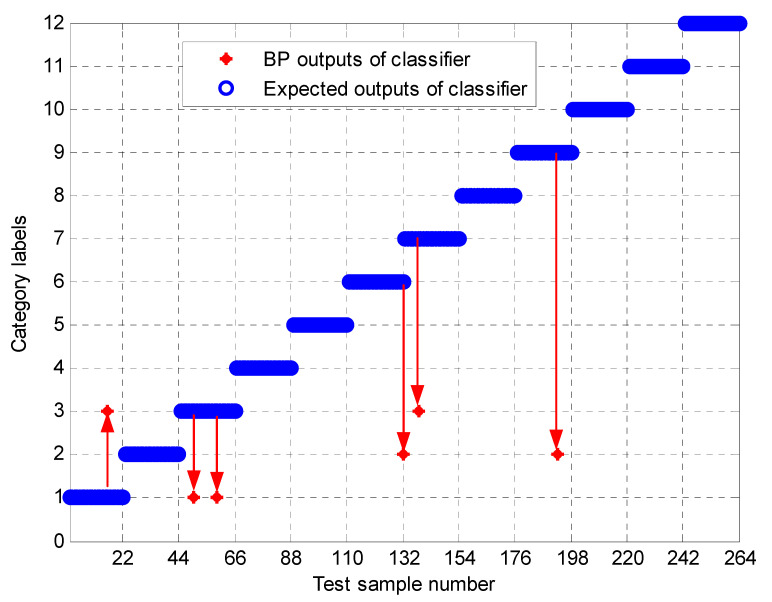
Output of Improved MPE based methods.

**Figure 20:**
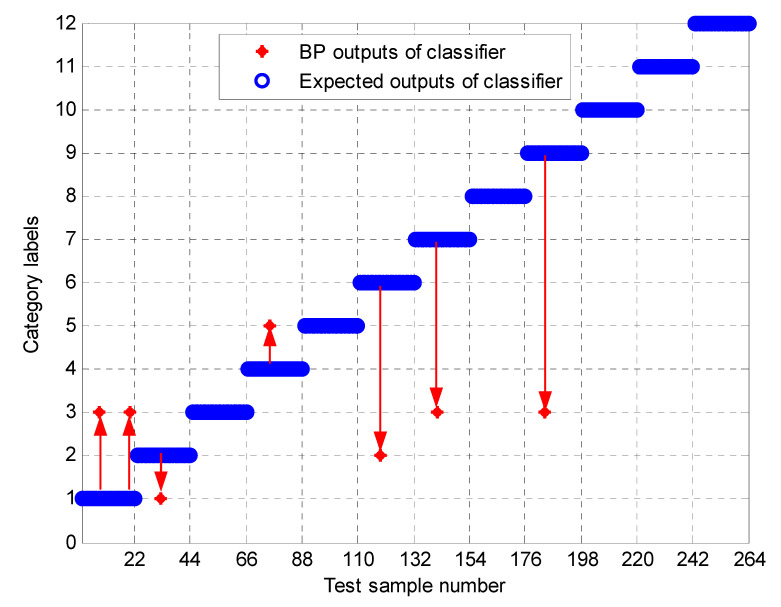
Output of EMD-Improved MPE-based methods.

**Figure 21:**
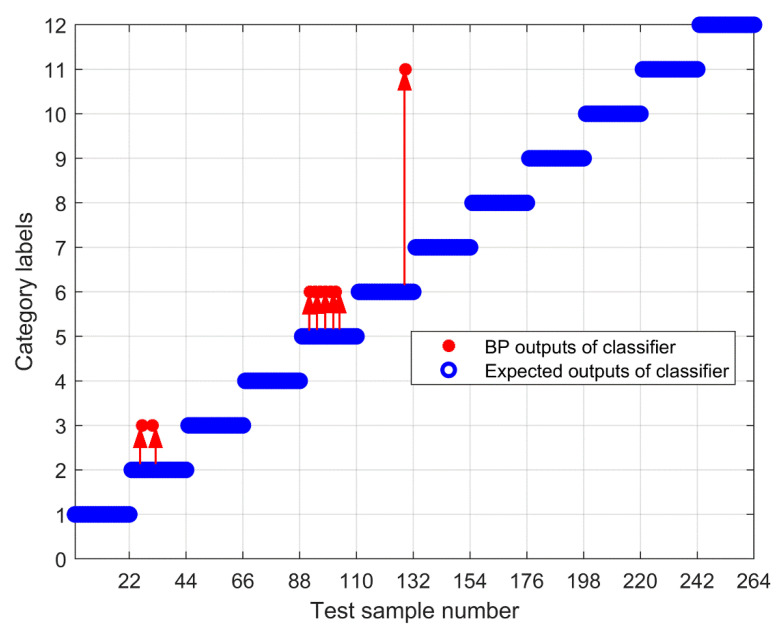
Output of EEMD-Improved MPE-based methods.

**Figure 22:**
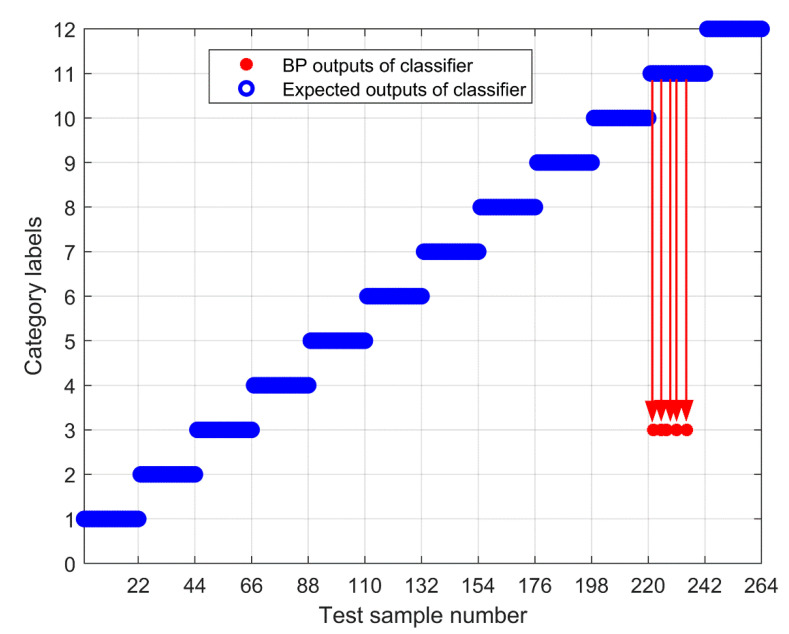
Output of IF-Improved MPE-based methods.

**Figure 23:**
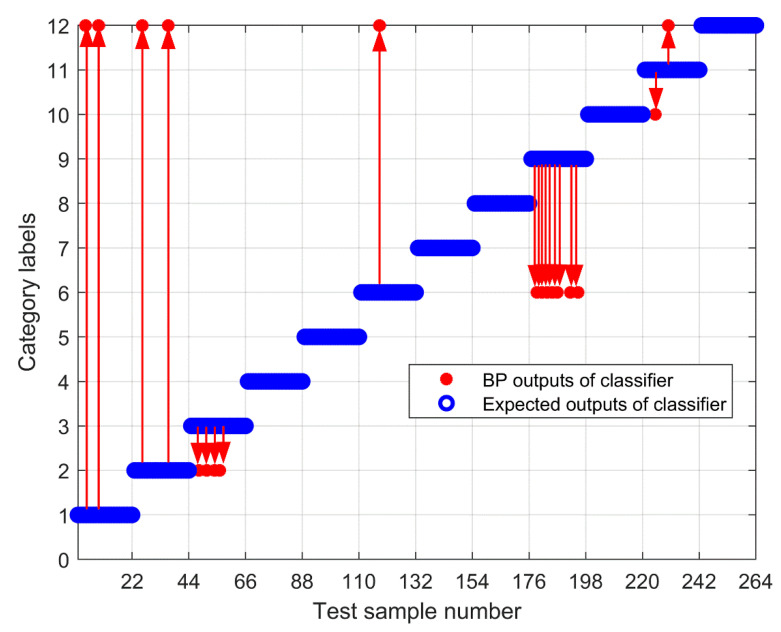
Output of LMD-Improved MPE-based methods.

**Figure 24:**
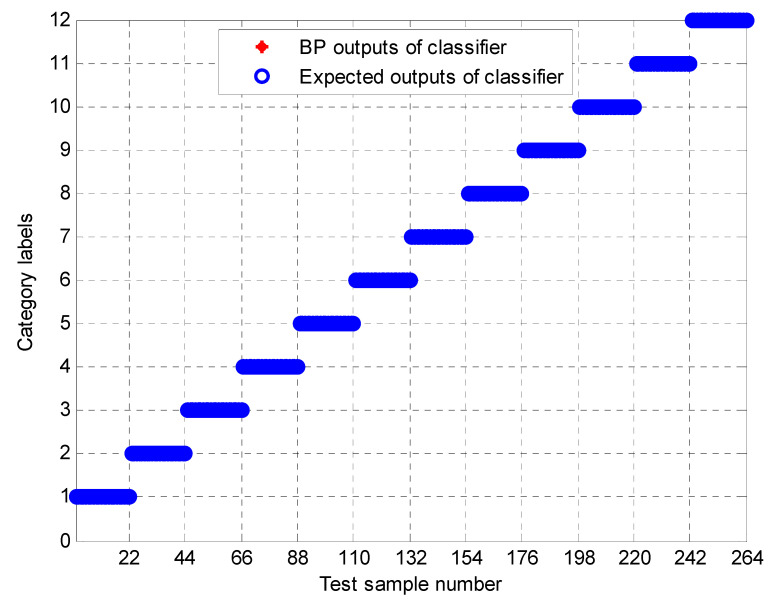
Output of k-Optimized ALIF and Improved MPE-based methods.

To further test the scheme in this paper, the MPE obtained above is input into the neural network as a feature vector, and the parameter selection of the BP neural network is shown in Table 3. To compare the performance of bearing fault identification. The results of performance testing on the original signal using MPE, Improved MPE, EMD-Improved MPE, EEMD-Improved MPE, IF-Improved MPE, LMD-Improved MPE, and the proposed fault identification scheme are shown in Figure 18, Figure 19, Figure 20, Figure 21, Figure 22, Figure 23 and Figure 24. Category labels 1 to 12 indicate different degrees of failure, and the corresponding relationship is shown in Table 2. It can be seen from the above results that using Improved MPE and MPE alone to directly extract the entropy value of the original signal as a feature vector for fault identification, Improved MPE has a higher accuracy than MPE, which shows that Improved MPE’s characterization ability is indeed better than MPE. Therefore, improved MPE is more suitable for extracting entropy characteristics of rolling bearing vibration signals under various conditions.

In addition, to avoid the randomness of the BP neural network, MPE-BP, improved MPE-BP, EMD-improved MPE-BP, EEMD-improved MPE-BP, IF-improved MPE-BP, LMD-improved MPE-BP, and k-optimized ALIF-improved MPE-BP methods were each randomly selected with different samples for training, and the remaining samples are tested. In this paper, this process is performed 50, and finally, the average of all results is taken as the final recognition result, as shown in Table 4. From Table 4, it can be seen that the k-optimized ALIF-improved MPE method requires the least training time and testing time, indicating that the proposed method in this paper can train a neural network with stable performance more quickly after feature extraction. In addition, the final average classification accuracy of k-optimized ALIF-improved MPE-BP for 50 times is as high as 99.98%, indicating that its classification reliability is better than that of MPE-BP, improved MPE-BP, EMD-improved MPE-BP, EEMD-improved MPE-BP, IF-improved MPE-BP, and LMD-improved MPE-BP methods The output of BP neural network shows that the scheme has good fault classification performance for bearings with different fault degrees and different fault categories.

## 6. Conclusions

This paper proposes a rolling bearing fault diagnosis scheme based on the k-optimized ALF, improved MPE, and BP neural network. Due to the theoretical limitations of the ALIF algorithm, it is necessary to select the number of decomposition layers according to experience. Therefore, the permutation entropy optimization algorithm is used to adaptively select the decomposition layer of ALIF, so that it can better decompose the vibration signal and obtain the optimal decomposition result. According to the coarse-graining process of MPE, the improved multi-scale permutation entropy is proposed. The results show that the improved multiscale permutation entropy has better stability and can more accurately characterize the complexity of the signal. The improved MPE of the optimal modal component is calculated and the fault feature vector is formed. The feature vector is input into the BP neural network to realize the fault pattern recognition. At the same time, to compare, the experimental signals are analyzed by using the improved MPE and the improved MPE based on EMD. The comparison results show that the scheme can effectively distinguish different types of faults and different degrees of faults with higher accuracy. However, it should be pointed out that the premise of all fault recognition rates is the vibration signal at the same rotational speed. Therefore, the next research focus is on the influence of rotation speed on fault recognition rate.

## Figures and Tables

**Table 1:** Experimental bearing parameters.

6205-2RS JEM SKF (Diameter/Inch)					
Ball Number	Contact Angle	Ball Diameter	Outside Diameter	Inside Diameter	Pitch Diameter
9	0	0.3126	2.0472	0.9843	1.537

**Table 2:** Experimental data for identification.

Fault Category	Fault Diameter	Label of Classification	Fault Category	Fault Diameter	Label of Classification
Ball Fault 1	0.007	1	Inner Race 3	0.021	7
Ball Fault 2	0.014	2	Inner Race 4	0.028	8
Ball Fault 3	0.021	3	Normal	0	9
Ball Fault 4	0.028	4	Outer Race 1	0.007	10
Inner Race 1	0.007	5	Outer Race 2	0.014	11
Inner Race 2	0.014	6	Outer Race 3	0.021	12

**Table 3:** Parameter selection of neural network.

Input Layer	Hidden Layer	Output Layer
12	10	12

**Table 4:** Average recognition rate of 50 runs for each classification method and neural network testing time.

Methods	Average Recognition Rate	Standard Deviation of Recognition Rate	Average Training Time	Average Testing Time
MPE	92.58%	0.03170	0.3011 s	1.0048 s
Improved MPE	96.25%	0.02990	0.2917 s	0.9438 s
EMD-Improved MPE	93.36%	0.03210	0.3068 s	1.0203 s
EEMD-Improved MPE	95.56%	0.03540	0.3100 s	1.0998 s
IF-Improved MPE	95.70%	0.03490	0.3198 s	1.0902 s
LMD-Improved MPE	91.57%	0.03399	0.3145 s	1.1282 s
K- ALIF- Improved MPE	99.98%	0.00079	0.2913 s	0.9213 s
